# Improvement of the bladder perfusion curative effect through tight junction protein degradation induced by magnetic temperature-sensitive hydrogels

**DOI:** 10.3389/fbioe.2022.958072

**Published:** 2022-08-04

**Authors:** Xiaoliang Sun, Xinhong Song, Peng Guo, Dong Zhang, Shishuai Zuo, Kang Leng, Yun Liu, Haiyang Zhang

**Affiliations:** ^1^ Department of Urology, Shandong Provincial Hospital Affiliated to Shandong First Medical University, Jinan, China; ^2^ Department of Logistics Management, Shandong Provincial Hospital Affiliated to Shandong First Medical University, Jinan, China; ^3^ Department of Urology, Shandong Provincial Hospital, Cheeloo College of Medicine, Shandong University, Jinan, China; ^4^ Knuppe Molecular Urology Laboratory, Department of Urology, School of Medicine, University of California, San Francisco, San Francisco, United States

**Keywords:** bladder perfusion, pirarubicin, chitosan, sustained release, tight junction, occludin

## Abstract

Postoperative intravesical instillation of chemotherapy is a routine procedure for non-muscular invasive bladder cancer (NMIBC). However, traditional bladder perfusion methods have insufficient exposure time, resulting in unsatisfactory therapeutic effects. In the present study, a chitosan (CS)-based *in situ* forming depot (ISFD) delivery system, including Fe_3_O_4_ magnetic nanoparticles (Fe_3_O_4_-MNP), CS, and β-glycerophosphate (GP) as main components, was synthesized. Pirarubicin (THP), as a chemotherapeutic drug, was loaded into the new system. Results showed that our carrier system (Fe_3_O_4_-THP-CS/GP) was converted into gel and attached to the bladder wall, possessing loose network structures with magnetic targeting and sustained release properties. Moreover, its retention time in bladder was more than 72 h accompanied by a suitable expansion rate and good degradation characteristics. The antitumor activities of Fe_3_O_4_-THP-CS/GP were more effective both *in vitro* and *in vivo* than the free THP solution. In the study of its mechanism, results showed that Fe_3_O_4_-THP-CS/GP suppressed the expression of occludin (OCLN) and affected tight junctions (TJ) between urothelial cells to promote THP absorption.

## 1 Introduction

Bladder cancer is one of the most common tumors in the urinary system. A total of 549,393 new cases and 199,922 deaths occurred in 2018 worldwide (Bray et al., 2018). NMIBC defined as the tumor confined to the mucosa and lamina propria of the mucosa accounts for about 80% of bladder cancers. Statistically, 50–70% of patients with NMIBC who undergo transurethral resection (TUR) surgery relapse within 5 years, among whom 20–30% will progress to a higher stage or develop metastases (Lehmann et al., 2006; Black et al., 2007).

Postoperative intravesical instillation of chemotherapy is a routine treatment for NMIBC to prevent recurrences. However, the recurrence rate will be reduced by only 14–17% by adding bladder instillation, without the inhibitory effect on tumor progression (Patel et al., 2015). Several limitations to traditional bladder perfusion should be noted. The blood–urine barrier, formed by the urothelium, can prevent the unregulated flow of toxins, ions, and water between urine and blood. Therefore, it is of fundamental importance for bladder function and metabolic homeostasis. But on the other hand, the blood–urine barrier seriously hindered the absorption of drugs and reduced the effect of bladder instillation (Kreft et al., 2010). Moreover, periodic urination led to insufficient exposure time (Parekh et al., 2006). Repeated instillations and increasing drug concentration will bring urethral injury, hematuria, and lower urinary tract symptoms to patients.

A variety of biomaterials even inorganic nanomaterials have been widely used in research to improve tumor therapy (Wang et al., 2020a; Wang et al., 2021; Xzab et al., 2021). We have been committed to ISFD to improve the efficacy of bladder instillation. ISFD is defined as a liquid formulation that, after entering the body, can be converted *in situ* to a solid or semisolid precipitated storehouse. The members of ISFD, bearing the characteristics of sustained release, can improve drug bioavailability and patients’ compliance. For instance, hydrogels prepared with lignin and graphene oxide have the ability to absorb impurities (Qian et al., 2020). Nanoparticle-loaded hydrogel had the ability to slow-release insulin and improve the pharmacological availability to 279.85% (Peng et al., 2013). In addition, the release rate of topotecan hydrochloride liposomes incorporated into the thermo-sensitive hydrogel was found to be slowed down (Xing et al., 2014).

We have tried to use a magnetic multiwalled carbon nanotube system to extend the duration of chemotherapeutic drugs in intravesical instillation by enhancing cytotoxicity and inhibiting cell proliferation (Suo et al., 2019). Moreover, we found the magnetic thermosensitive chitosan/β-glycerophosphate (CS/GP) hydrogel as a feasible matrix for drug delivery, which permitted an intravesical continuous release of the drug over 48 h, resulting in enhancing the antitumor efficacy (Zhang et al., 2014; Sun et al., 2016). CS is a suitable component of ISFD due to its good biocompatibility and non-toxicity (Madni et al., 2021). CS itself had no detrimental effect on urothelial cancer cells. One of the possible mechanisms of this delivery system for enhancing therapeutic effects was that CS increased the permeability of the urothelial layer, broke TJ, and reduced transepithelial resistance. More importantly, CS enabled the complete recovery of the permeability barrier after 24 h (Višnjar et al., 2017). This phenomenon was also observed in intestinal epithelial cells, leading to increased drug absorption (Rosenthal et al., 2012). CLDN4 protein, a transmembrane protein associated with TJ, might be one underlying effector regulated by CS (Yeh et al., 2011).

In the present study, a CS-based ISFD delivery system, including Fe_3_O_4_-MNP, CS, and GP as main components, was synthesized to examine its targeting and sustained release capability. Meanwhile, antitumor efficacies *in vivo* and *in vitro* and underlying mechanisms of this system loaded with the chemotherapeutic agents were also explored.

## 2 Results

### 2.1 Characterization of Fe_3_O_4_-THP-CS/GP

**FIGURE 1:**
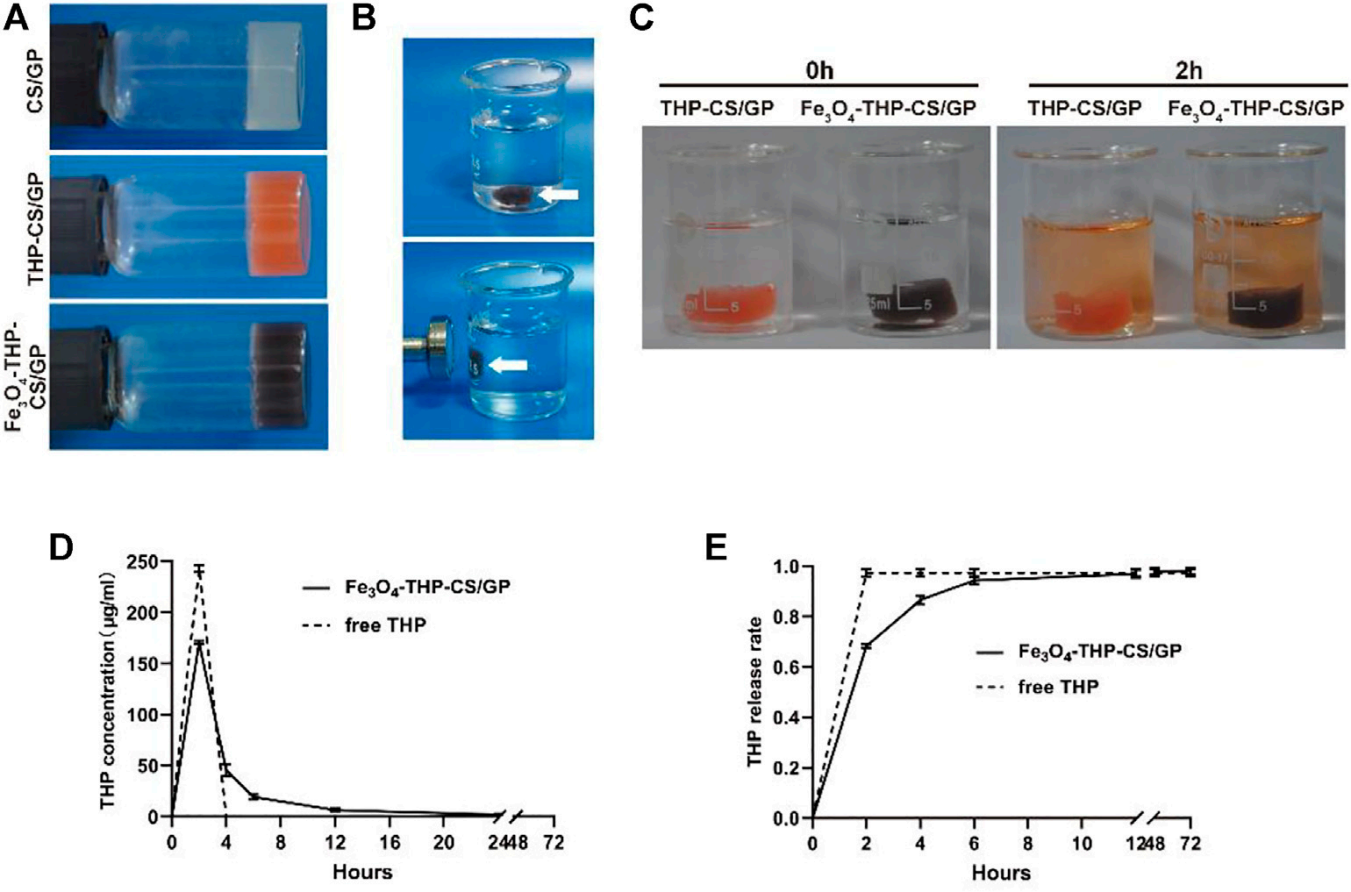
Appearance and characteristics of Fe_3_O_4_-THP-CS/GP. **(A)** Liquid gel changes into a solid and stably sticks to the wall of the container. CS/GP gel is milky white, orange with THP, and black with Fe_3_O_4_-MNP. **(B)** When the external magnetic field was applied, the Fe_3_O_4_-THP-CS/GP gel at the bottom of the container was quickly adsorbed to the sidewall, demonstrating the gel’s good magnetic targeting performance. **(C)** Color of the medium containing THP-CS/GP and Fe_3_O_4_-THP-CS/GP gradually changed to orange in 2 h (the color of THP solution is orange), which showed sustained release performance of the gel. **(D)** Drug concentration curve in the medium of Fe_3_O_4_-THP-CS/GP and free THP solution. **(E)** Cumulative release rate curve of Fe_3_O_4_-THP-CS/GP and free THP solution.

Fe_3_O_4_-THP-CS/GP prepared in the present study showed its transformation ability from liquid to gel in 3.5 min (Figure 1A), which was important for bladder perfusion. Moreover, it had good magnetic targeting performance, allowing quick adherence to the target within 1 s (Figure 1B). In the dissolution experiment, the medium color of THP-CS/GP and Fe_3_O_4_-THP-CS/GP gradually displayed orange even after several changes in the dissolution medium within 24 h (Figure 1C). The release of THP from the drug delivery system was slow, with a weaker burst peak (Figure 1D, 170.81 ± 1.78 μg/ml vs 243.32 ± 3.61 μg/ml) and a longer release time (Figure 1E, approximately 12 vs 2 h) compared with the free THP solution, indicating the sustained release of THP from the delivery system.

**FIGURE 2:**
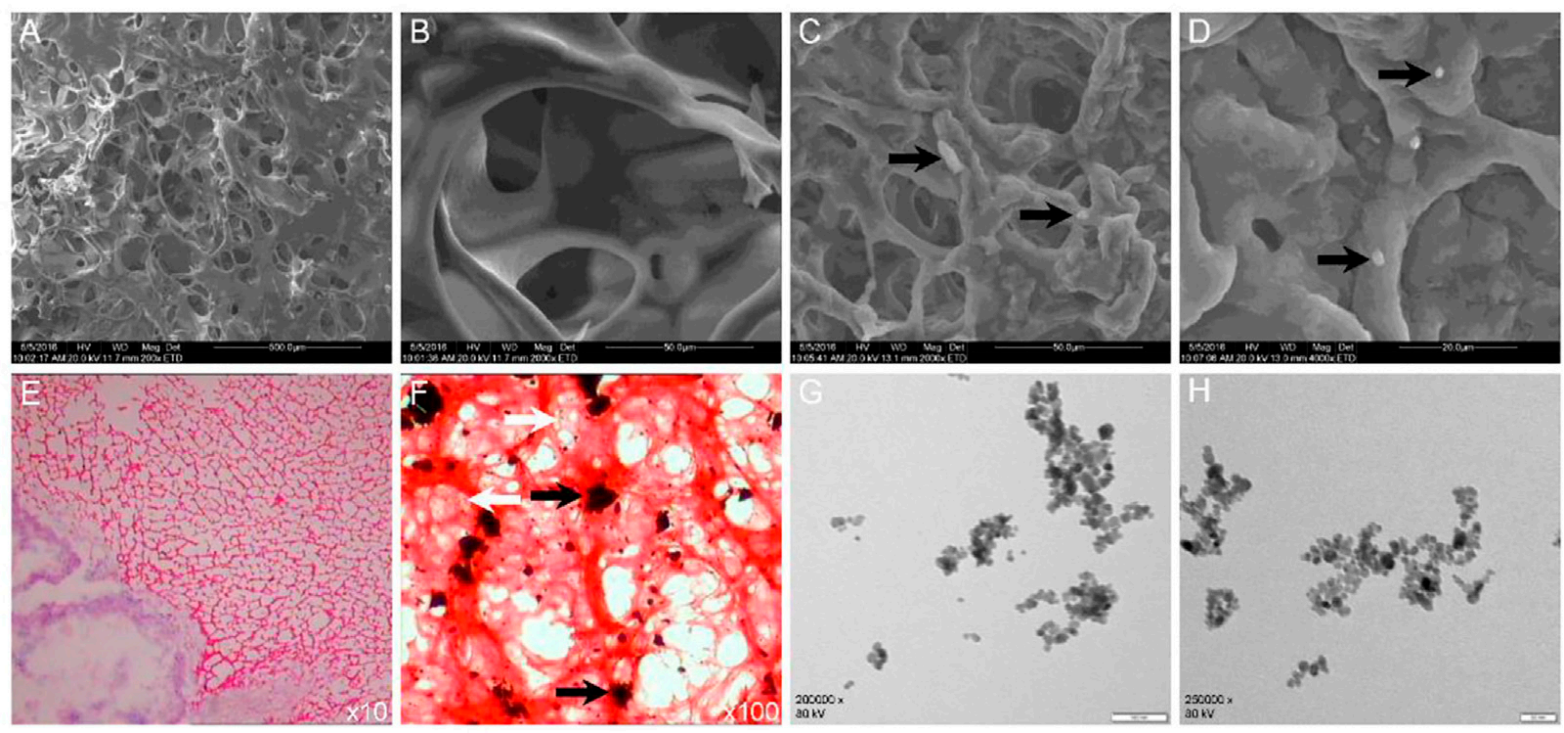
Microstructure of CS/GP and Fe_3_O_4_-THP-CS/GP. **(A**,**B)** Microstructure of the CS/GP gel by SEM. **(C**,**D)** Microstructure of the Fe_3_O_4_-THP-CS/GP gel obtained by SEM. The THP granules were indicated by black arrows. **(E**,**F)** Microstructure of the Fe_3_O_4_-THP-CS/GP gel obtained by HE staining. The THP and Fe_3_O_4_-MNP granules were marked by white and black arrows, respectively. **(G**,**H)** Transmission electron microscopic images of Fe_3_O_4_-MNP at different magnifications.

The main characteristic of the CS/GP gel under a scanning electron microscope (SEM) was porous and smooth, while the outer surface of the Fe_3_O_4_-THP-CS/GP gel was rough and granular due to THP and Fe_3_O_4_-MNP stuck to or embedded in the gel matrix (Figure 2). Hematoxylin-eosin (HE) staining showed Fe_3_O_4_-THP-CS/GP as a mesh network in freezing conditions, with Fe_3_O_4_-MNP and THP particles embedded in a matrix.

### 2.2 Swelling and degradation characteristics of Fe_3_O_4_-THP-CS/GP

**FIGURE 3:**
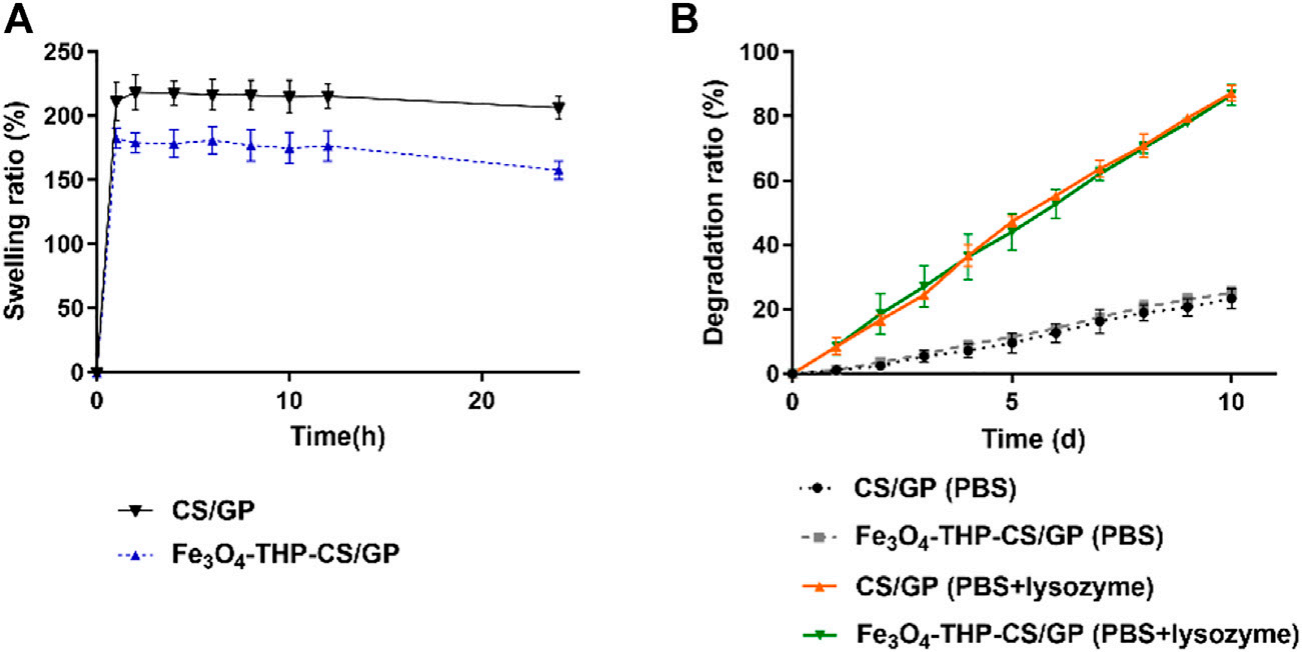
Line chart of swelling and the degradation rate of different gels. **(A)** CS/GP and Fe_3_O_4_-THP-CS/GP gel had good swelling properties, and the swelling equilibrium was reached in about 1 h (211.50% ± 15.00% vs. 182.50% ± 8.10%; *p* < 0.05), but the swelling property of Fe_3_O_4_-THP-CS/GP was significantly lower than that of CS/GP. **(B)** Degradation of CS/GP and Fe_3_O_4_-THP-CS/GP gels in pure PBS was slow, and the degradation rate was less than 30% in 10 days. After lysozyme was added, the degradation rate of the two was significantly accelerated. The degradation rate of different solvents was significantly different on the 10th day (23.30% ± 3.08% vs. 87.17% ± 2.55%, *p* < 0.0001 for CS/GP; 25.13% ± 1.94% vs. 86.65% ± 3.21%, *p* < 0.0001 for Fe_3_O_4_-THP-CS/GP).

The swelling data are shown in Figure 3A. CS/GP and Fe_3_O_4_-THP-CS/GP gels swelled fast and reached the equilibrium swelling after 1 h incubation in PBS, with a swelling ratio of 211.50% ± 15.00% vs. 182.50% ± 8.10% (*p* < 0.05). The difference was statistically significant.

The CS/GP and Fe_3_O_4_-THP-CS/GP gels showed similar degradation curves in the same degradation medium (Figure 3B). With the different media, Fe_3_O_4_-THP-CS/GP in PBS degraded slowly with only 25.13% ± 1.94% after 10 days. But its degradation rate in enzyme solution is up to 86.65% ± 3.21%. There was a significant statistical difference between the two degradation rates (*p* < 0.0001). The CS/GP gel had the same result.

### 2.3 Antitumor activity *in vitro*

**FIGURE 4:**
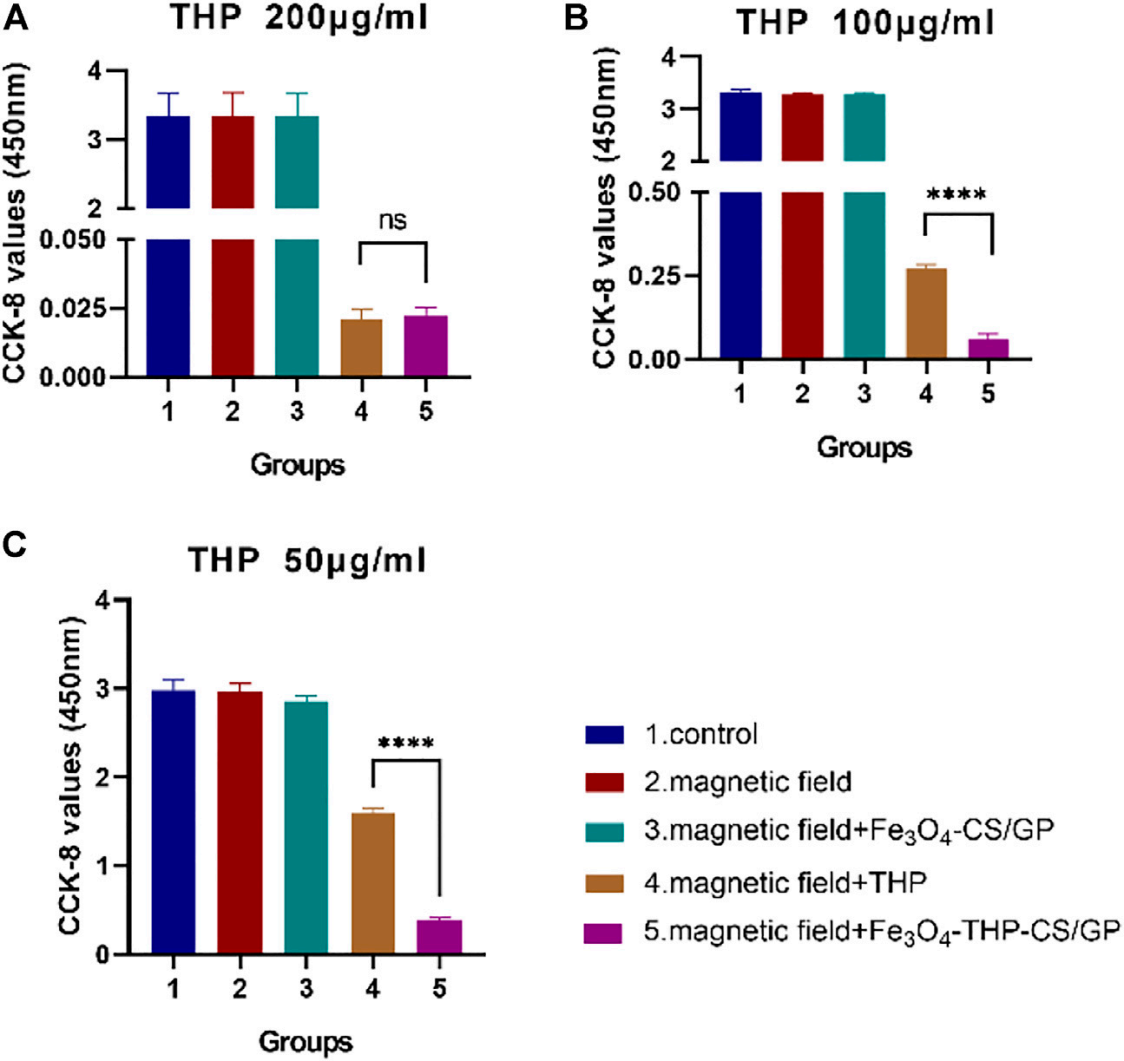
CCK-8 values were examined to determine of the antitumor activity of Fe3O4-THP-CS/GP *in vitro* with different THP concentrations **(A)** 200 µg/ml **(B)** 100 µg/ml **(C)** 50 µg/ml (*p* < 0.0001).

No significant differences in CCK-8 values among the control group, magnetic field group, and magnetic field + Fe_3_O_4_-CS/GP group were observed (the data are shown in Table 1) in three groups of different concentrations of THP treatment (Figure 4). CCK-8 values were dramatically suppressed by adding THP (200 μg/ml) both in the magnetic field + THP group and magnetic field + Fe_3_O_4_-THP-CS/GP group but without significant difference (0.021 ± 0.0036 vs. 0.023 ± 0.0023; *p* > 0.9999). However, CCK-8 values were significantly lower in the magnetic field + Fe_3_O_4_-THP-CS/GP group than the magnetic field + THP group whether THP was 100 μg/ml (0.272 ± 0.01 vs 0.061 ± 0.016; *p* < 0.0001) or 50 μg/ml (1.603 ± 0.049 vs 0.381 ± 0.034; *p* < 0.0001).

**TABLE 1:** *p* values of the cell activity among the first three groups of different THP concentrations (1: control group; 2: magnetic field group; and 3: magnetic field + Fe_3_O_4_-CS/GP group).

Concentration	1v2	1v3	2v3
THP 200 μg/ml	*p* > 0.9999	*p* > 0.9999	*p* > 0.9999
THP 100 μg/ml	*p* = 0.0689	*p* = 0.1682	*p* = 0.9733
THP 50 μg/ml	*p* = 0.9998	*p* = 0.2818	*p* = 0.3494

### 2.4 Retention characteristic of Fe_3_O_4_-THP-CS/GP in the bladder

**FIGURE 5:**
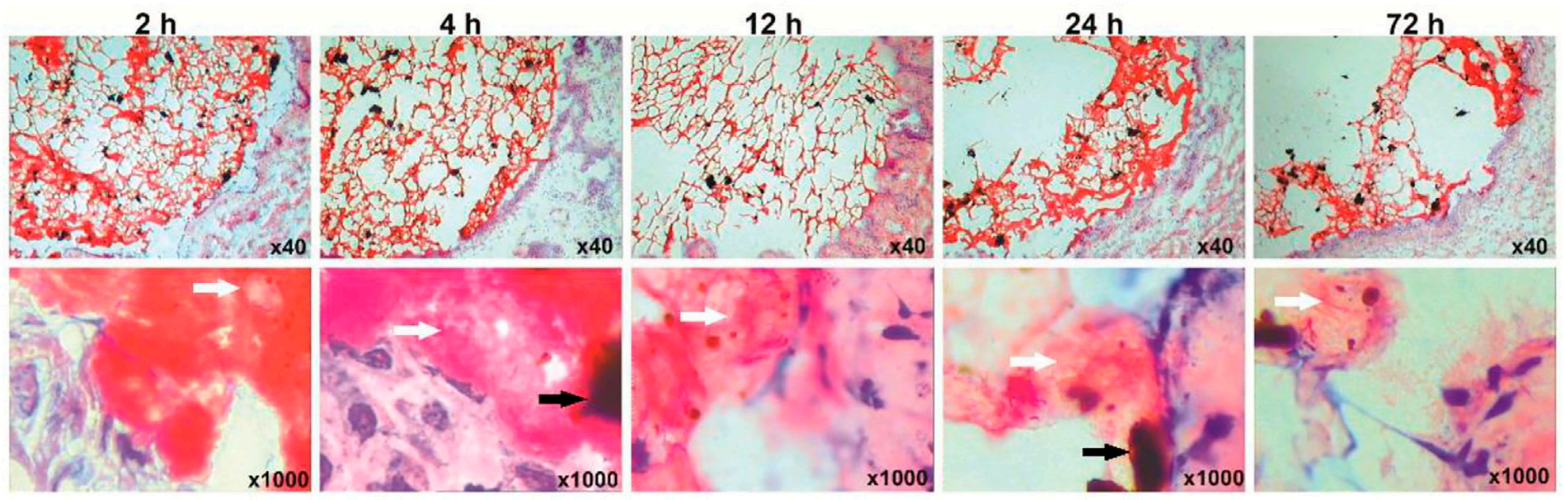
Retention of the Fe_3_O_4_-THP-CS/GP gel on the bladder wall at different time points. The black and white arrows marked Fe_3_O_4_-MNP and THP granules, respectively.

Fe_3_O_4_-THP-CS/GP solution was immediately converted to gel and stuck to the bladder wall after perfusion for 2 h (Figure 5). The mass of the gel degraded gradually with the extension of observation time. Magnetic gel and THP particles were still observed after bladder perfusion for 72 h.

### 2.5 Antitumor ability *in vivo*

**FIGURE 6:**
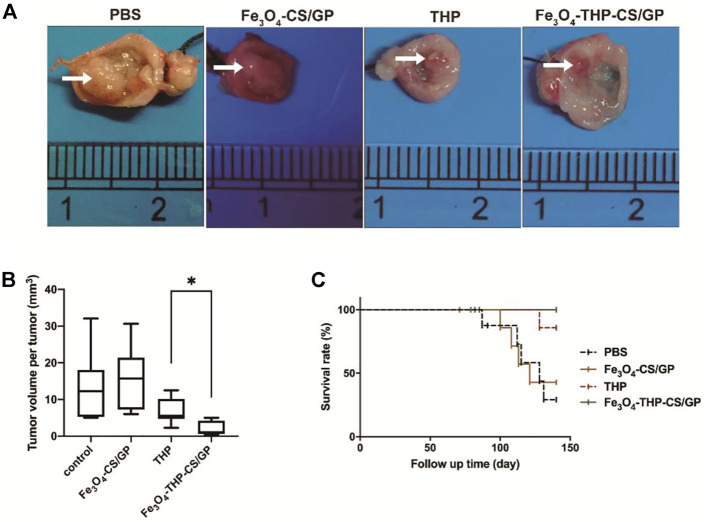
*In vivo* antitumor activity. **(A)** Representative images of bladder tumors in each treatment group. Relatively large bladder tumors are shown in groups treated with PBS and Fe_3_O_4_-CS/GP. Small tumors are observed in the group treated with free THP solution. The smallest tumors without progression are found in the group treated with Fe_3_O_4_-THP-CS/GP. **(B)** Summary of the tumor volume per tumor data. **(C)** Survival rates of the different groups upon tumor induction, *p* < 0.05. The white arrows indicate the tumors (*p* < 0.05).

Representative bladder tumors are shown in Figure 6A. The tumors in rats that received PBS and Fe_3_O_4_-CS/GP were larger with wider bases and obvious infiltrative growth. Tumor volumes in rats that received THP and Fe_3_O_4_-THP-CS/GP were smaller than the other rats. Moreover, significantly smaller tumors were observed in the Fe_3_O_4_-THP-CS/GP group than in the THP group (Figure 6B, 2.125 ± 1.976 mm^3^ vs 6.863 ± 3.716 mm^3^; *p* < 0.05). The survival rate is a decisive parameter to evaluate the antitumor efficacy in animal experiments. Kaplan–Meier analysis showed that the Fe_3_O_4_-THP-CS/GP treatment group had the highest survival rate and the longest survival time (Figure 6C, *p* < 0.05).

### 2.6 Mechanism of CS-mediated TJ opening

**FIGURE 7:**
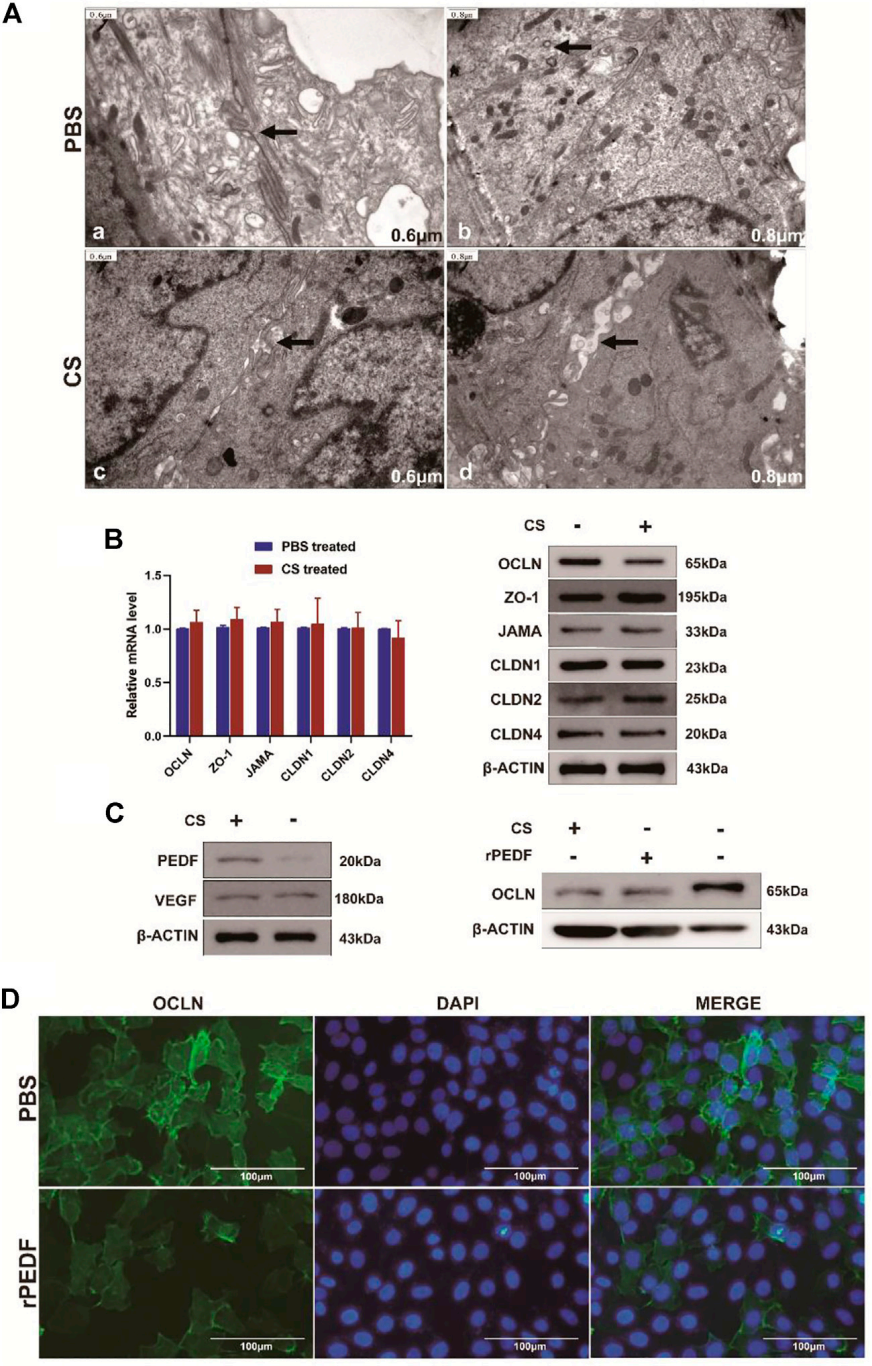
Changes in the TJ structure and related protein expression after CS treatment. **(A)** a, b after PBS treatment, the two cells remained closely attached, and the TJ structure was intact; c, d after CS induction, gaps appeared between adjacent cells, and TJ was opened. **(B)** Changes in TJ-related genes at transcription and translation levels after CS treatment. **(C)** After CS treatment, the PEDF expression increased, while the VEGF expression did not change significantly. After CS or rPEDF treatment, the OCLN expression was significantly reduced. **(D)** Fluorescence intensity of OCLN decreased obviously after being treated with rPEDF.

As can be seen in Figure 7Aa, b, before CS treatment, the adjacent bladder tumor cells were closely connected with each other under a transmission electron microscope (TEM). TJ between two cells appeared as two closed parallel lines without space between them. After CS treatment, the intercellular space was looser and wider than before (Figure 7Ac, d), losing the intact structures.

Six common TJ-related genes were analyzed, namely, *OCLN*, *ZO-1*, *JAMA*, *CLDN1*, *CLDN2*, and *CLDN4*. There were no significant changes in the transcription level of these genes after CS treatment in bladder tumor cells (Figure 7B, Supplementary Table S1, *p* > 0.05). According to Western blot analysis, the expression level of OCLN protein was significantly decreased after CS treatment (*p* < 0.05), but the expression levels of the other TJ proteins did not change significantly (Figure 7B, Supplementary Figure S1A).

Western blot results showed the expressions of the pigment epithelium-derived factor (PEDF) in T24 cells were significantly upregulated by adding CS (*p* < 0.05), but the expression of VEGF did not change significantly (Figure 7C, Supplementary Figure S1B, *p* > 0.05). The expressions of OCLN protein were significantly decreased by adding CS (*p* < 0.001) or exogenous recombinant PEDF (rPEDF) (Figure 7C, Supplementary Figure S1C, *p* < 0.01). Moreover, immunofluorescence staining showed the fluorescence intensities of OCLN in the bladder tumor cells were significantly reduced after being treated with rPEDF (Figure 7D).

## 3 Discussion

CS/GP hydrogels are composed of three-dimensional polymer networks with numerous hydrophilic cavities. They are pliable, viscoelastic, and compatible with most living tissues, making no damage to surrounding tissues when implanted into the host (Bhattarai et al., 2010). Hence, CS/GP hydrogels are ideal candidates for biomedical applications, such as tissue repair and drug delivery. We previously found that the deacetylation degree of CS affected gel transformation time, while the molecular weight of CS and loaded drugs had no such effect (Sun et al., 2016).

In the present study, the significant difference in the swelling rate between CS/GP and Fe_3_O_4_-THP-CS/GP is mainly due to the partial space occupied by Fe_3_O_4_-MNP and THP. But this does not affect Fe_3_O_4_-THP-CS/GP possessing good swelling performance. The CS/GP and Fe_3_O_4_-THP-CS/GP showed similar degradation curves, implying that neither Fe_3_O_4_-MNP nor THP affected the degradation property. Moreover, the Fe_3_O_4_-THP-CS/GP gel degraded slowly and lasted more than 10 days in the enzyme solution. Different from the results *in vitro*, the retention study demonstrated that degradation was significantly faster *in vivo*. The main reason is a variety of enzymes contained in the urine. Second, the washing effect of urine will also accelerate the disintegration of the gel. In general, the degradation ratio of Fe_3_O_4_-THP-CS/GP in the harsh environment of the bladder was excellent. The holes and space were smaller in Fe_3_O_4_-THP-CS/GP, reflecting the embedding of THP and Fe_3_O_4_-MNP in the gels. Moreover, the fast swelling and easy irrigation characteristics of our system indicated good liquid absorption and sustained release of THP. On the other hand, the swelling ratio of Fe_3_O_4_-THP-CS/GP could be adjusted by regulating the amounts of THP and Fe_3_O_4_-MNP.

In the traditional bladder perfusion therapy, the drug burst release demonstrated an intense one-time pattern. Some patients cannot bear bladder irritation and thus urinate early, leading to unsatisfactory therapeutic effects (Chen et al., 2019), while the release of THP from Fe_3_O_4_-THP-CS/GP gel was gentle, with a weaker initial burst and release for a longer time even after several times of micturition. THP was embedded or attached to the gel matrix, and it could only be released after the solvent penetration or after the gel gradually degraded. In addition, the interactions between drug and matrix molecules also contributed to the sustained release. THP and CS both contain amino groups that form hydrogen bonds (Teodori et al., 2019), which enhance the mutual attraction and promote sustained release. Chemical modifications can further optimize this effect. For instance, a mixture of biodegradable nanoparticles and CS/β-glycerophosphate gel showed an enhanced ability to deliver and release insulin slowly (Peng et al., 2013), by which only 19.11% of total insulin was released within 31 days. However, most formulations and chemically modified CS-based gel carriers were prepared using organic solvents and chemical crosslinkers. Clinical applications were restricted by their toxicities and complex preparations (Rizeq et al., 2019; Cao et al., 2022). In our research, Fe_3_O_4_-THP-CS/GP had the advantages of simple preparation, low cost, and non-toxicity to achieve the goals of sustained release.

No significant differences in CCK-8 values among the control group, magnetic field group, and magnetic field + Fe_3_O_4_-CS/GP group (Figure 4) was observed, prompting that Fe_3_O_4_-CS/GP and magnets had made no difference in the drug action of THP. With the decrease in the THP dose, the antitumor effect of the magnetic field + Fe_3_O_4_-THP-CS/GP group was significantly stronger than that of the free THP group. This indicates that the prolonged exposure time and sustained-release properties of Fe_3_O_4_-THP-CS/GP can enhance the antitumor activity of THP. From the microscopic structure of the Fe_3_O_4_-THP-CS/GP gel, the porous and loose structure provided a channel for medium penetration, allowing the drug to dissolve and release slowly.

The amino group of CS is positively charged, that is, it exhibits cationic properties. The negatively charged (anionic) acidic groups in the mucosa attract the CS through ion interaction to achieve adhesion (Grabovac et al., 2005). The interactions between CS and mucosa were improved by increasing the temperature, deacetylation degree of CS, and keeping the medium pH value between the isoelectric point (2.6) of mucin and the ionic dissociation constant (6.5) in CS (Lang et al., 2020). In the present study, normal body temperature, pH of urine, and 95% deacetylation of CS were in proper conditions to achieve a better adhesion effect.

As narrated earlier, prolonged gel retention time was one of the reasons for better antitumor effects. Another reason that should be considered is that CS could increase drug absorption by affecting TJ. TJ, the main structure of the blood–urine barrier of bladder, effectively prevents the metabolic wastes and toxic substances in the urine from being reabsorbed through the urothelium (Lewis, 2000; Ma et al., 2018). Moreover, with the increase in tissue depth, the drug content decreased by about 50% every 500 μm (Wientjes et al., 1993). The drug content in the urothelium and lamina propria was about 2.8% of that in the bladder lumen. Therefore, normal TJ can prevent THP absorption in traditional bladder perfusion. CS was found not only to remove the glycosaminoglycan layer on the mucosal surface of bladder but also to open the TJ to promote the absorption of moxifloxacin (Kerec et al., 2005). Another report showed CS promoted the transport of alginic acid and polyglucuronic acid by reversibly opening TJ (Wang et al., 2020b). This conclusion was also verified by the result in the present study, which was the opening of the TJ structure after CS treatment. Moreover, OCLN, one of TJ-related proteins, was detected to express less in the present study. Although ubiquitination and degradation of OCLN induced by VEGF can reduce the functions of TJ (Murakami et al., 2009; Li et al., 2019), its regulatory effect on TJ and cell permeability remains to be elucidated. However, it was found in our study that the VEGF expression did not change after CS treatment. Therefore, we hypothesized that CS might induce OCLN degradation through other pathways.

PEDF, an endogenously secreted glycoprotein, is the most powerful angiogenesis inhibitor discovered at present, which can inhibit angiogenesis by inhibiting VEGF. Hence, a higher expression of PEDF was associated with less metastasis and better prognosis in patients with tumors (Rivera-Perez et al., 2018). In the present study, PEDF was significantly upregulated by adding CS, inducing the suppression of OCLN. How PEDF regulates OCLN remains unclear. p38/MAPK, the downstream pathway of PEDF in the process of cancer cachexia, can upregulate a variety of E3 ubiquitin ligases in tumor cells, leading to ubiquitination and degradation of proteins (Zhang et al., 2011; Quan-Jun et al., 2017; Mulder et al., 2020). In addition, polychlorinated biphenyls can promote Itch expression by activating the p38/MAPK pathway (Jia et al., 2017). Interestingly, Itch was an E3 ubiquitin ligase that induced ubiquitination degradation of OCLN (Konson et al., 2018), which needs further research for confirmation.

## 4 Materials and methods

### 4.1 Materials

CS powder (419419; >75% deacetylation, Mw 310–375 kDa, viscosity 800–2000 cP) was purchased from Sigma-Aldrich (St. Louis, MO, United States ). THP (IP1500) was purchased from Solarbio Life Sciences Co., LTD. (Beijing, China). Analytical-grade GP (G9422) was acquired from Sigma-Aldrich (St. Louis, MO, United States ). Fe_3_O_4_-MNP (N106282027275; >99.5% purity, 50 nm) was obtained from Changsha Jingkang Co., LTD. (Hubei, China). The exogenous recombinant PEDF (SRP4988) was purchased from Thermo Fisher Technology Co., LTD. (Shanghai, China). Anti-β-actin (ab8227), anti-VEGF (ab32152), anti-PEDF (ab14993), anti-OCLN (ab216327), anti-ZO-1 (ab276131), anti-JAMA (ab52647), anti-CLDN1 (ab211737), anti-CLDN2 (ab53032), and anti-CLDN4 (ab210796) antibodies were obtained from Abcam (Shanghai) Trading Co., LTD. (Shanghai, China). N-butyl-N-(4-hydroxybutyl) nitrous amide (BBN) (B8601) was acquired from Sigma-Aldrich (St. Louis, MO, United States ). Superscript III reverse transcriptase (18080044) was purchased from Invitrogen Co., LTD. (Carlsbad, CA, United States ). The chemiluminescent HRP substrate (P90719) was acquired from Millipore Corporation (Billerica, MA, United States ). Secondary antibodies Alexa Fluor 594 goat anti-rabbit (B40925), TRI reagents (AM9738), and DAPI (D3571) were purchased from Thermo Fisher Technology Co., LTD. (Shanghai, China). All other chemicals and reagents used in this study were of analytical grade.

Seventy-six 8-week-old female Wistar rats were maintained in a temperature and humidity-controlled room on 12-h light/dark cycles. All rats had free access to water and food. All animal care, treatments, and procedures in this study were approved by the Experimental Animal Ethics Committee of Provincial Hospital affiliated to Shandong First Medical University (No. 2020–171), and animal care followed National Institutes of Health criteria for the care and use of laboratory animals in research.

### 4.2 Preparation and basic characteristics of the Fe_3_O_4_-THP-CS/GP system

#### 4.2.1 Preparation of the Fe_3_O_4_-THP-CS/GP gel

CS/GP solution was prepared according to the previous method (Zhang et al., 2013). Briefly, the CS powder was dissolved in 0.1 M hydrochloric acid and then stirred magnetically at room temperature for 2 h. GP powder was dissolved in distilled water. Both solutions were cooled in an ice bath for 10 min. The GP and CS were then mixed under magnetic agitation at 4°C to form a clear and uniform solution. Fe_3_O_4_-MNP (W/V: 0.3%) and THP powder (W/V: 0.1%, unless otherwise specified) were successively added to CS/GP mixture under stirring and then dispersed by ultrasound.

#### 4.2.2 Sustained-release characteristics of Fe_3_O_4_-THP-CS/GP

The comparisons of the drug release behavior between the Fe_3_O_4_-THP-CS/GP gel system and traditional bladder perfusion were analyzed by the dialysis method (Peng et al., 2010). The drug loading gel was placed in a dialysis bag and then placed in a beaker containing the dissolution medium. The dissolution medium was changed every 2 h in a 37°C constant temperature water bath shaker to simulate the urination cycle. The collected dissolution samples were stored at −80°C after filtration for batch testing. The concentration of THP was determined by high-performance liquid chromatography (HPLC) (Yi et al., 2015). After statistical data, the concentration–time curve and the total amount of release were plotted. The peak concentration and release time were compared with traditional bladder perfusion.

#### 4.2.3 Magnetic targeting characteristics of Fe_3_O_4_-THP-CS/GP

The magnetic targeting ability of the Fe_3_O_4_-THP-CS/GP system was also tested. A clot of the Fe_3_O_4_-THP-CS/GP gel was laid on the bottom of a beaker filled with phosphate buffer solution. Then, a magnet was placed outside the beaker to observe the movement of Fe_3_O_4_-THP-CS/GP.

#### 4.2.4 Observation of internal structures of the Fe_3_O_4_-THP-CS/GP system

After gelation, CS/GP and Fe_3_O_4_-THP-CS/GP were lyophilized and sprayed with gold to increase the electrical conductivity. The internal structure of CS/GP and Fe_3_O_4_-THP-CS/GP was observed and analyzed under SEM (Hitachi, SU-70, Japan) at an acceleration voltage of 3–20 kV. Moreover, a total of 0.1 ml of the Fe_3_O_4_-THP-CS/GP mixture was sectioned and stained by HE to observe the internal structures under a digital microscope.

### 4.3 Examinations of the swelling rate and degradation rate of Fe_3_O_4_-THP-CS/GP

The swelling rate and degradation rate of Fe_3_O_4_-THP-CS/GP were examined by gravimetric analysis.

CS/GP and Fe_3_O_4_-THP-CS/GP gels were prepared, freeze-dried, and weighed. Dry weight (*W*0) was recorded. The xerogel was placed in 10 ml PBS at pH 6.0 and heated in water bath at 37°C. At the time points of 1, 2, 4, 6, 8, 10, 12, and 24 h, the gels were carefully removed, the water attached to the surface of the gels was gently wiped off with a filter paper, and the wet weights of the gels were recorded as *W*t. The swelling rate was calculated by the following formula. To generate statistically relevant data, three independent parallel experiments were repeated.

$$\text{Swelling rate }=\;\frac{W\text{t}-W0}{W0}\;\times \;100\text{\%}\text{.}$$

The degradation rate was tested following similar protocols. The degradation solution was a pH 6.0 PBS solution containing 4 mg/ml lysozyme. PBS without lysozyme was used as control. The xerogel prepared earlier was placed in the degradation solution in a 37 °C water bath shaker (60 rpm). At the set time points of 1, 2, 3, 4, 5, 6, 7, 8, 9, and 10 days, the gels were carefully removed and washed with distilled water. After vacuum drying, the dry weight was recorded as *W*d. The degradation rate was calculated by the following formula. Parallel experiments were repeated three times.

$$\text{Degradation rate }=\;\frac{W0-W\text{d}}{W0}\;\times \;100\text{\%}\text{.}$$

### 4.4 Examinations of the antitumor activity of Fe_3_O_4_-THP-CS/GP *in vitro*

The CCK-8 kit was used for detection. After 96-well plate inoculation, T24 cells were grouped and given different treatments: the blank control group without special treatment; external magnetic field; external magnetic field + Fe_3_O_4_-CS/GP gel; external magnetic field + THP solution (200, 100, and 50 μg/ml, respectively); and external magnetic field + Fe_3_O_4_-THP-CS/GP gel (the same dose of THP as mentioned previously). The gel was attracted to the sidewall using a magnetic field to avoid pressing the cells. The medium was changed every 2 h to simulate periodic urination. After treatment for 24 h, a CCK-8 reagent was added to measure the optical density (OD).

### 4.5 Retention capacity of Fe_3_O_4_-THP-CS/GP in the bladder

A fresh liquid solution of Fe_3_O_4_-THP-CS/GP was prepared. Self-made 3F catheters were inserted into the bladders of 27 female Wistar rats. A measure of 0.1 ml of the Fe_3_O_4_-THP-CS/GP solution was infused into each bladder. The rats were then placed in a cage surrounded by a magnetic field of 0.4 T. At each time point of 2, 4, 6, 8, 10, 12, 24, 48, and 72 h, three rats were sacrificed. Their bladders were collected. HE staining was performed to observe the retention of Fe_3_O_4_-THP-CS/GP at different time points after perfusion.

### 4.6 Examinations of the antitumor activity of Fe_3_O_4_-THP-CS/GP *in vivo*

**FIGURE 8:**
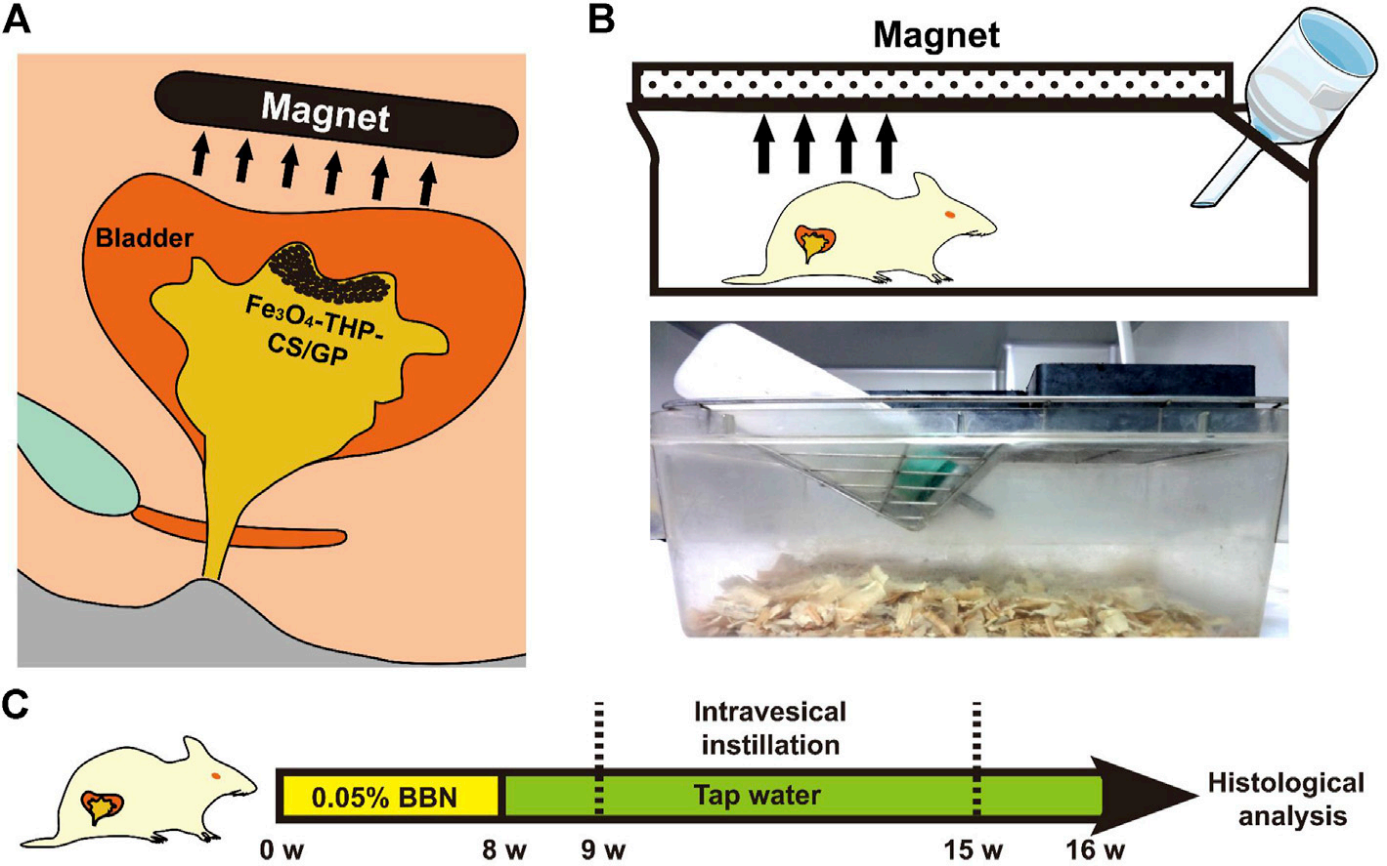
Schematic diagram of the *in vivo* antitumor activity of Fe_3_O_4_-THP-CS/GP. **(A)** Schematic diagram of the clinical application of Fe_3_O_4_-THP-CS/GP. **(B)** Schematic illustration of the rat terrarium. A magnet above the terrarium attracts Fe_3_O_4_-THP-CS/GP to the bladder wall. **(C)** Animal treatment protocol.

The experimental procedure is shown in Figure 8. Thirty-two female Wistar rats were randomly divided into four groups. All rats were fed with normal diets supplemented with 0.05% BBN in tap water at the tumorigenesis stage for 8 weeks. From the 10th week, all rats were entered into a bladder perfusion period. The rats in the first group received PBS instillation, the second group received Fe_3_O_4_-CS/GP instillation, the third group received THP instillation (THP 0.25 mg), and the fourth group received Fe_3_O_4_-THP-CS/GP instillation (THP 0.25 mg). The volume of the perfusion solution for each rat was 0.1 ml once a week for 6 weeks. The rats of the second and fourth groups were kept in an external magnetic field of 0.4 T. All rats were evaluated periodically. Time of death, hematuria, and weight loss were recorded. Autopsies were carried out immediately if rats died. After 20 weeks, all the rats were sacrificed to collect bladders, ureters, and kidneys. The size and number of tumors in the bladders of each group were compared. Survival data were estimated by the Kaplan–Meier method. The Mantel–Cox log-rank test was used for statistical analysis.

### 4.7 Examinations of the effect of CS on TJ

#### 4.7.1 Transmission electron microscopy detection

Urothelial carcinoma-bearing rats prepared earlier were perfused with CS into the bladder for 1 h. The bladder mucosal tissues were collected, washed by PBS, fixed in 3.7% paraformaldehyde, fixed in osmium tetroxide, dehydrated using gradient alcohol baths (25%, 50%, 75%, and 100%), embedded in Spurr resin, and polymerized at 70°C. Ultra-thin slices were made with a diamond knife, stained with toluidine blue, and observed under an optical microscope. The sections with toluidine blue staining areas were then loaded onto a TEM grid and examined using a Philips CM10 electron Optics B.V. apparatus at 120 kV accelerating voltage. TJ structures between adjacent cells were identified.

#### 4.7.2 Real-time PCR analysis

Monolayer T24 cells were treated with CS to study the effect of CS on expressions of TJ genes. Total RNA was isolated using TRI reagents, according to the manufacturer’s instructions. It was then reverse transcripted into cDNA using random hexamers and superscript III reverse transcriptase. The Power SYBR Green PCR Master Mix was used with the Applied Biosystems 7,500 real-time fluorescent quantitative PCR system. There were three copies per sample and per gene. The primers used were as follows (Table 2).

**TABLE 2:** Primers of TJ genes for PCR.

Gene name	Abbreviation	Positive (5’→3′)	Reverse (5’→3′)
Occludin	OCLN	ACA​AGC​GGT​TTT​ATC​CAG​AGT​C	GTC​ATC​CAC​AGG​CGA​AGT​TAA​T
Zona occludens 1	ZO-1	TTGGCGAGAAACGCTATG	TTG​GCA​GAA​GAT​TGT​GAT​TG
Junctional adhesion molecule A	JAMA	AAG​GAG​ACA​CCA​CCA​GAC​T	AGGCATCACTATCCCATC
Claudin-1	CLDN1	ACA​GCA​TGG​TAT​GGC​AAT​AGA​ATC​G	GGG​ACA​GGA​ACA​GCA​AAG​TAG​GG
Claudin-2	CLDN2	GCC​TCT​GGA​TGG​AAT​GTG​CC	GCT​ACC​GCC​ACT​CTG​TCT​TTG
Claudin-4	CLDN4	AGA​GTG​GAT​GGA​CGG​GTT​TAG​AGG	TGAAGCGGGTGAGCAGAG
Glyceraldehyde-3-phosphate dehydrogenase	GAPDH	GCA​CAG​TCA​AGG​CCG​AGA​AT	GCC​TTC​TCC​ATG​GTG​GTG​AA

#### 4.7.3 Protein extraction and Western blotting

Total protein was extracted to analyze the effect of CS on TJ expressions. After CS and exogenous recombinant PEDF treatment, T24 monolayer cells were gently washed twice with ice PBS and then covered with lysate buffer for 30 min. After centrifugation at 10,000 rpm for 5 min, the supernatant was collected for subsequent Western blot analysis. The protein concentration was calculated by the Bradford method. Equivalent protein samples were separated on 12% SDS-polyacrylamide gel and then transferred to nitrocellulose membranes. After blocking in 5% skimmed milk in Tris buffer brine containing 0.05% Tween-20 (TBST), the membrane was incubated overnight in a 4 °C blocking buffer containing a diluted primary antibody, including anti-OCLN, anti-ZO-1, anti-JAMA, anti-CLDN1, 2, 4, anti-VEGF, and anti-PEDF. Subsequently, the membrane was washed three times with TBST and then exposed to appropriate alkaline phosphatase-coupled secondary antibodies, followed by observation using a chemiluminescent HRP substrate. ImageJ software (National Institute of Health, Bethesda, MD, United States ) was used to perform density analysis for specific bands.

#### 4.7.4 Immunofluorescence staining

T24 cells grown on culture plates were incubated for 30 min with CS before processing for confocal microscopy. Cells were rinsed with PBS, fixed with methanol, and permeabilized with PBS containing 0.5% Triton X-100. The primary antibody employed was anti-OCLN, and the secondary antibody that we used was Alexa Fluor 594 goat anti-rabbit. DAPI was used to stain cell nuclei. Fluorescence images were obtained with a confocal microscope (LSM 510 meta, Carl Zeiss, Jena, Germany).

### 4.8 Statistical analyses

All experiments were repeated three times, and the results are presented as the mean ± standard deviation (SD). Statistical significance was determined by one-way ANOVA, Student’s *t*-test, and Mantel–Cox log-rank test, as appropriate, using GraphPad Prism 8 (Dotmatics, San Diego, CA, United States ). A *p*-value <0.05 was considered significant, whereas *p* > 0.05 was considered to be non-significant.

## 5 Conclusion

In this study, we prepared a novel drug delivery system by loading THP with the CS thermosensitive gel. After perfused into the bladder, the Fe_3_O_4_-THP-CS/GP mixture was converted into the gel and attached to the bladder wall. Due to sustained release and targeting properties, Fe_3_O_4_-THP-CS/GP was proved to possess better antitumor effects by affecting the TJ function. This result was achieved by enhancing the PEDF expression and modification of OCLN after using CS.

## Data Availability

The original contributions presented in the study are included in the article/Supplementary Material; further inquiries can be directed to the corresponding author.
