# Supplementary material for: Improvement of the bladder perfusion curative effect through tight junction protein degradation induced by magnetic temperature-sensitive hydrogels
Source: Front Bioeng Biotechnol. 2022 Aug 4;10:958072. doi: 10.3389/fbioe.2022.958072 (PMC9386042; doi:10.3389/fbioe.2022.958072)
Supplement: Supplementary file 1 [file DataSheet1.docx]

**Table S1.** After CS treatment, there was no significant difference in TJ-related gene expression at the transcriptional level.

|  | Significantly? | *P* value | Mean of PBS treated | Mean of CS treated |
| --- | --- | --- | --- | --- |
| OCLN | No | 0.420699 | 1.005 | 1.063 |
| ZO-1 | No | 0.308196 | 1.017 | 1.093 |
| JAMA | No | 0.469077 | 1.011 | 1.066 |
| CLDN1 | No | 0.797821 | 1.009 | 1.047 |
| CLDN2 | No | 0.979074 | 1.007 | 1.010 |
| CLDN4 | No | 0.400718 | 1.003 | 0.9155 |


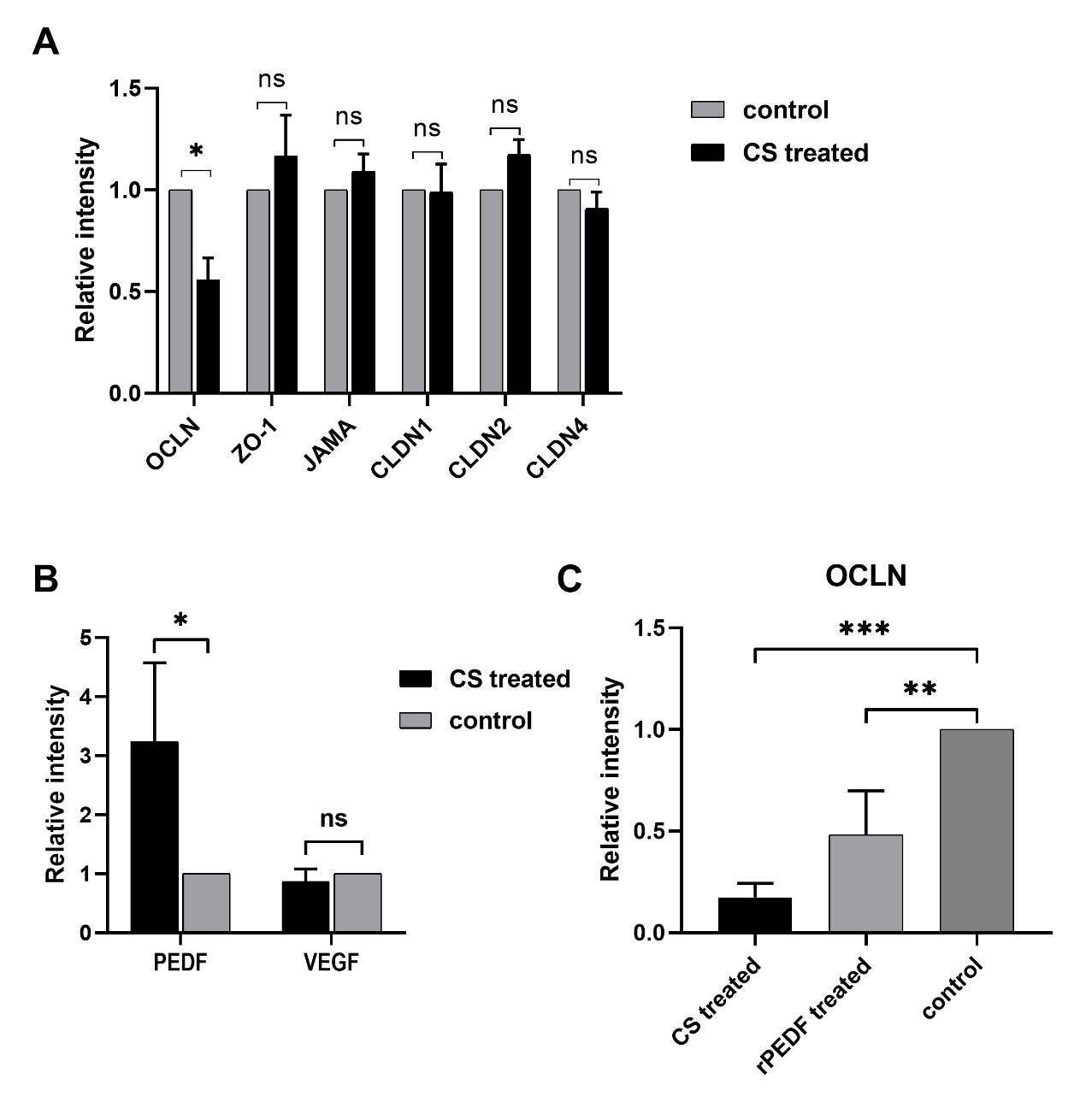


**Figure S1.** Quantitative histogram of CS regulation on TJ-related protein expression. (A) After CS treatment, the expression level of OCLN protein was significantly decreased (*P* < 0.05), but the expression levels of the other TJ proteins did not change significantly. (B) After CS treatment, the expressions of PEDF were significantly up-regulated (*P* < 0.05), but there was no statistical difference in VEGF expression. (C) After CS or rPEDF treatment, the expressions of OCLN protein were significantly decreased. (^🞷^*P* < 0.05, ^🞷🞷^*P* < 0.01 ^🞷🞷🞷^*P* < 0.001, ^🞷🞷🞷🞷^*P* < 0.0001)
